# Six-month stability and predictive validity of the personality inventory for ICD-11

**DOI:** 10.1186/s40359-022-00979-2

**Published:** 2022-11-16

**Authors:** Johannes Stricker, Louisa Jakob, Denis Köhler, Reinhard Pietrowsky

**Affiliations:** 1 Psychology Department, Heinrich Heine University Düsseldorf, Universitätsstraße 1, 40225 Düsseldorf, Germany; 2 Faculty of Social Sciences and Cultural Studies, University of Applied Sciences Düsseldorf, Düsseldorf, Germany

**Keywords:** Personality inventory for ICD-11, Predictive validity, Stability, Psychological distress

## Abstract

**Background:**

The International Classification of Diseases 11th Revision (ICD-11) personality disorder model comprises, among other elements, five maladaptive personality trait domains (negative affectivity, detachment, dissociality, disinhibition, anankastia). Recently, the personality inventory for ICD-11 (PiCD) has emerged as one of the most widely used measures of these ICD-11 personality trait domains.

**Methods:**

The current study contributed to the validation of the PiCD validation by exploring its stability and predictive links with psychological distress over 6 months in a sample of 206 German community adults.

**Results:**

The PiCD trait domain scales displayed strong differential (all *r* ≥ .80) and absolute stability (all |*d|* ≤ .09). Additionally, PiCD negative affectivity predicted depression, anxiety, and stress, and PiCD detachment predicted depression over 6 months beyond baseline.

**Conclusion:**

In sum, this study demonstrated the stability of the PiCD trait domain scores, supporting their utility for capturing relatively stable traits as described in the ICD-11. Additionally, we provided the first evidence for the predictive validity of some of the PiCD trait domain scores.

**Supplementary Information:**

The online version contains supplementary material available at 10.1186/s40359-022-00979-2.

## Background

The International Classification of Diseases 11th Revision (ICD-11) introduces a novel personality disorder model [1]. This model comprises a rating of general personality disorder severity, differentiating personality difficulty, mild, moderate, and severe personality disorder. Additionally, practitioners may further describe a person’s personality pathology using a borderline pattern specifier or any number of five personality trait domain specifiers (negative affectivity, detachment, dissociality, disinhibition, anankastia). Different self-report scales exist for assessing personality disorder severity [2, 3] and the ICD-11 personality trait domains. The personality inventory for ICD-11 [4] has recently emerged as one of the most frequently used self-report measures of the ICD-11 personality trait domains. Hence, many studies have investigated this instrument’s validity and psychometric soundness (e.g., [5, 6]). Yet, the stability of the PiCD trait domain scores over multiple months and their predictive validity are, thus far, unclear. Stability and predictive validity are crucial features of assessments of maladaptive personality. Thus, we addressed these research gaps by investigating the stability and predictive links of the PiCD with psychological distress over six months in a general population sample.

### The personality inventory for ICD-11 (PiCD)

The PiCD is a 60-item self-report instrument assessing the five ICD-11 personality trait domains with 12 items each. The PiCD has been validated in general population and clinical samples and has been translated into various languages (e.g., [6, 7]). In previous studies, the PiCD has displayed strong internal consistencies and meaningful correlations with other measures of maladaptive personality (e.g., [4]).

### The stability of the PiCD trait domain scores

The ICD-11 describes personality as “an individual’s *characteristic* [emphasis added] way of behaving, experiencing life, and of perceiving and interpreting themselves, other people, events, and situations” [1]. Additionally, personality disorder is characterized as being “relatively stable after young adulthood” [1]. Thus, although personality disorder may exacerbate or remit over the life course (see [8]), measures of the ICD-11 personality trait domains should display a substantial degree of stability over several months.

Stability over time may be evaluated by the stability of the relative positioning of a person within a reference sample (differential stability) and as the average change observed in a sample overall (absolute stability) [9]. Previous research on established measures of maladaptive personality typically revealed substantial differential and temporal stability (e.g., [10]). Thus far, only one study has used the PiCD over more than one measurement point [6]. In this study, all PiCD trait domain scores displayed large test-retest reliabilities (*r* = .81 to .89) in a sample of Italian community adults.

### The predictive validity of the PiCD trait domain scores

The ICD-11 states that personality disorder is associated with “substantial distress or significant impairment” [1]. Hence, several studies have used psychological distress scales, such as the Depression Anxiety Stress Scales-21 (DASS-21 [11]), to validate the PiCD (e.g., [12]). Among the PiCD trait domain scales, particularly negative affectivity, but also detachment, and disinhibition, were substantially linked to psychological distress (e.g., [5]). However, to date, links between the PiCD trait domain scores and psychological distress have only been assessed cross-sectionally. Thus, methodological artifacts, such as content overlap at item-level, could explain the identified associations. Hence, besides stability, this study aimed to assess the predictive validity of the PiCD trait domain scores.

## Materials and methods

### Procedure and sample

We recruited German-speaking community adults through various social media channels, websites, and flyers. The participants completed the PiCD and assessments of psychological distress online at two measurement points, spaced six months apart (*M* = 178 days, *SD* = 14.35). As compensation for participation, participants could receive an individual personality profile or course credit. To detect careless responders, we used two instructed response items at each measurement point. Six participants were excluded for failing to answer these items correctly.

The final sample comprised 206 German community adults (165 female, 37 male, three other, one non-disclosure) with a mean age of 27.54 years (*SD* = 12.02, range = 18 to 84). Sixty-three participants (30.58%) indicated holding a university degree, 142 (68.93%) had a secondary school degree, and one indicated not having completed secondary education. Sixty-two participants (30.10%) were employed or self-employed, 131 (63.59%) were students or trainees, and 13 (6.31%) were currently not working (e.g., retired). We did not determine the sample size a priori, but we terminated data collection after a predefined period of one month at both measurement points. All participants provided their informed consent.

### Measures

*Personality Inventory for ICD-11 (PiCD).* We used the German version of the PiCD [4, 7], a self-report measure of the ICD-11 personality trait domains. The PiCD assesses each ICD-11 personality trait domain with 12 items, using a 5-point Likert scale ranging from 1 “strongly disagree” to 5 “strongly agree”. Various studies attest to the reliability and validity of the German PiCD in community adults (e.g., [13]).

*Depression Anxiety Stress Scales-21 (DASS-21).* We used the German version of the DASS-21 [11, 14] to assess three psychological distress domains (depression, anxiety, stress) with seven items each. Participants indicated their experienced symptoms over the past week on a 4-point Likert scale ranging from 0 “Did not apply to me at all” to 3 “Applied to me very much or most of the time”. Various studies support the reliability and validity of the German DASS-21 for capturing psychological distress in community adults (e.g., [15]).

### Statistical analyses

We estimated the 6-month differential stability of the PiCD scores by computing the bivariate correlations between each personality trait domain score at T1 and T2. Additionally, we estimated the 6-month absolute stability by comparing the T1 and T2 scores for each personality trait domain in a series of paired sample *t*-tests. Finally, we evaluated the predictive validity of the PiCD trait domain scores in a series of hierarchical regressions. In each regression, one T2 measure of psychological distress was predicted by its baseline score (T1) in Step 1 and, additionally, by one T1 PiCD trait domain score (Step 2). The data and code are available via the Open Science Framework: https://osf.io/gy2d6/?view_only=17db78478cff4f22927df10bdd4d9abb.

## Results

### Preliminary analyses

Table S1 in Additional file 1 shows the bivariate correlations, descriptive statistics, and reliabilities for all variables. The PiCD trait domain scales and the DASS-21 subscales displayed satisfactory reliabilities (Cronbach’s α = .74 to .91, McDonald’s ω = .74 to .91). Bivariately, T1 negative affectivity correlated with T1 depression (*r* = .54, 95% CI [.44, .63], *p* < .001), T1 anxiety (*r* = .58, 95% CI [.48, .67], *p* < .001), and T1 stress (*r* = .65, 95% CI [.58, .72], *p* < .001). T1 detachment correlated with T1 depression (*r* = .38, 95% CI [.26, .50], *p* < .001) and T1 anxiety (*r* = .16, 95% CI [.03, .29], *p* = .019). Additionally, T1 dissociality (*r* = .20, 95% CI [.06, .33], *p* = .004) and T1 disinhibition (*r* = .17, 95% CI [.03, .30], *p* = .017) correlated with T1 stress. Finally, T1 anankastia correlated with T1 depression (*r* = .16, 95% CI [.02, .29], *p* = .024), T1 anxiety (*r* = .16, 95% CI [.03, .29], *p* = .019), and T1 stress (*r* = .16, 95% CI [.02, .29], *p* = .026).

### Six-month stability of the PiCD trait domain scores

The PiCD trait domain scores displayed large autocorrelations between the two measurement points, *r* = .81, 95% CI [.76, .85] for negative affectivity, *r* = .87, 95% CI [.83, .90] for detachment, *r* = .81, 95% CI [.75, .85] for dissociality, *r* = .80, 95% CI [.74, .84] for disinhibition, and *r* = .81, 95% CI [.75, .85] for anankastia (all *p*s < .001), indicating strong differential stability. Regarding absolute stability, the PiCD trait domain scales displayed only insubstantial mean-level change, *t*(205) = .23, *p* = .817, *d* = .02 for negative affectivity, *t*(205) = − 1.33, *p* = .185, *d* = − .09 for detachment, *t*(205) = − .44, *p* = .660, *d* = − .03 for dissociality, *t*(205) = .34, *p* = .732, *d* = .02 for disinhibition, and *t*(205) = − .87, *p* = .386, *d* = − .06 for anankastia.

### Predictive validity of the PiCD trait domain scores

Tables S2 to S6 in Additional file 1 display the full results of the hierarchical regressions. T1 negative affectivity significantly predicted T2 depression (β = .14, 95% CI [.01, .27], *p* = .030, ∆*R*^*2*^ = .01), T2 anxiety (β = .16, 95% CI [.03, .30], *p* = .021, ∆*R*^*2*^ = .02), and T2 stress (β = .21, 95% CI [.06, .36], *p* = .007, ∆*R*^*2*^ = .03) beyond baseline. T1 detachment significantly predicted T2 depression (β = .12, 95% CI [.003, .23], *p* = .045, ∆*R*^*2*^ = .01), but not T2 anxiety (β = .06, 95% CI [− .06, .17], *p* = .316, ∆*R*^*2*^ = .00) or T2 stress (β = .06, 95% CI [− .06, .18], *p* = .304, ∆*R*^*2*^ = .00) beyond baseline. T1 dissociality predicted neither T2 depression (β = − .03, 95% CI [− .14, .08], *p* = .557, ∆*R*^*2*^ = .00), nor T2 anxiety (β = − .05, 95% CI [− .17, .06], *p* = .363, ∆*R*^*2*^ = .00) nor T2 stress (β = .03, 95% CI [− .09, .15], *p* = .625, ∆*R*^*2*^ = .00) significantly beyond baseline. Similarly, T1 disinhibition did not significantly predict T2 depression (β = .06, 95% CI [− .05, .17], *p* = .272, ∆*R*^*2*^ = .00), T2 anxiety (β = .05, 95% CI [− .06, .17], *p* = .375, ∆*R*^*2*^ = .00), or T2 stress (β = .11, 95% CI [− .01, .23], *p* = .073, ∆*R*^*2*^ = .00) beyond baseline. Finally, T1 anankastia did not significantly predict T2 depression (β = − .03, 95% CI [− .14, .08], *p* = .563, ∆*R*^*2*^ = .00), T2 anxiety (β = .00, 95% CI [− .11, .12], *p* = .988, ∆*R*^*2*^ = .00), or T2 stress (β = − .06, 95% CI [− .18, .06], *p* = .338, ∆*R*^*2*^ = .00) beyond baseline.

## Discussion

### Current findings

This study investigated the stability and predictive validity of the PiCD over six months in German community adults. All PiCD trait domain scores displayed strong differential and absolute stability. The differential stability coefficients were large and almost identical to test–retest correlations obtained over two weeks in previous work on the PiCD (i.e., .80 ≥ *r* ≤ .89 [6]). Thus, this study provided the first evidence that the PiCD captures relatively enduring traits rather than fluctuations in symptomology or distress. These findings corroborate the notion that maladaptive personality traits are relatively stable, also compared to general personality disorder severity [16]. This point is further supported by the finding that the PiCD stability coefficients were notably larger (*r* = .80 to .87) than those of the DASS-21 scales (*r* = .52 to .64, see Additional file 1: Table S1).

Regarding predictive validity, this study showed that the PiCD negative affectivity and detachment scores explain future psychological distress beyond baseline. Thus, these PiCD trait domain scales appear to capture maladaptive tendencies predisposing to increases in psychological distress over a relatively short period of six months. Similar prospective links have recently been reported for other personality traits. In a study with three independent prospective samples, personality traits predicted first onsets of mental disorders, symptom chronicity, and functioning beyond prior and current mental disorder diagnoses [17]. Together, these findings attest to the utility of personality traits for incrementally predicting future psychological distress and psychopathology.

In line with previous studies on maladaptive personality traits (e.g., [18]), the PiCD trait domain scores were differentially related to psychological distress in our study. As in prior work on neuroticism (e.g., [19]), the predictive links identified in this study were particularly pronounced for negative affectivity, supporting its central role in developing psychopathology and distress. Interestingly, other cross-sectional associations (e.g., of dissociality and disinhibition with stress) did not translate into predictive relations beyond baseline. This finding highlights the importance of carefully distinguishing between cross-sectional associations and predictive links when investigating personality-psychological distress associations.

As we used a non-clinical sample, only preliminary implications for clinical practice can be derived from this study. Given the identified stability coefficients, it appears justifiable for clinicians to interpret and communicate the PiCD scores as indicators of a patient’s relatively stable behavioral, cognitive, and affective patterns. Additionally, the identified predictive links show that the PiCD negative affectivity and detachment scores may help identify patients with a heightened risk of increasing psychological distress over the following months.

### Limitations and future research

This study has some limitations, pointing to crucial future research directions. First, this study covered a relatively short period (six months) and only two measurement points. Further research on the stability and predictive utility of the PiCD using longer time intervals and more measurement points is needed. Second, we used a predominantly female general population sample. Although using preselected (e.g., clinical) samples may lead to an underestimation of stability coefficients due to regression to the mean [9], further research in clinical settings is required. Additionally, larger and more diverse samples are needed to establish whether this study’s findings replicate across different subpopulations. Third, this study relied on self-report measures. Future studies are needed to evaluate the stability and prognostic validity of the informant/clinician-report version of the PiCD [5, 20]. Fourth, in this study, we used three relatively broad indicators of psychological distress. Future research is needed to test more specific predictive relations (e.g., of detachment and loneliness).

## Conclusion

Taken together, this study supports the PiCD trait domain scores’ utility for assessing relatively stable maladaptive personality characteristics. Additionally, we provided the first evidence for the predictive validity of the PiCD, but more research using longer time intervals and more specific outcomes is needed.

## Supplementary Information

**Additional file 1:** Supplementary Analyses.

## Data Availability

The dataset and code supporting the conclusions of this article are available via the Open Science Framework: https://osf.io/gy2d6/?view_only=17db78478cff4f22927df10bdd4d9abb.

## Abbreviations

DASS-21: Depression Anxiety Stress Scales-21
ICD-11: International Classification of Diseases 11th Revision
PiCD: Personality Inventory for ICD-11
WHO: World Health Organisation
