# Supplementary material for: Six-month stability and predictive validity of the personality inventory for ICD-11
Source: BMC Psychol. 2022 Nov 16;10:270. doi: 10.1186/s40359-022-00979-2 (PMC9670366; doi:10.1186/s40359-022-00979-2)
Supplement: Supplementary file 1 — Additional file 1: Supplementary Analyses. [file 40359_2022_979_MOESM1_ESM.pdf]

# Supplementary Material

## Six-Month Stability and Predictive Validity of the Personality Inventory for ICD-11

Table S1

*Bivariate Correlations, Means (M), Standard Deviations (SD), and Reliabilities for all Variables*

| Variable | 1 | 2 | 3 | 4 | 5 | 6 | 7 | 8 | 9 | 10 | 11 | 12 | 13 | 14 | 15 | 16 |
|---|---|---|---|---|---|---|---|---|---|---|---|---|---|---|---|---|
| 1. Negative affectivity T1 | - | | | | | | | | | | | | | | | |
| 2. Detachment T1 | .29$^{***}$ | - | | | | | | | | | | | | | | |
| 3. Dissociality T1 | .00 | .10 | - | | | | | | | | | | | | | |
| 4. Disinhibition T1 | -.02 | -.02 | .41$^{***}$ | - | | | | | | | | | | | | |
| 5. Anankastia T1 | .41$^{***}$ | .40$^{***}$ | -.15$^{*}$ | -.59$^{**}$ | - | | | | | | | | | | | |
| 6. Depression T1 | .54$^{***}$ | .38$^{***}$ | .05 | .11 | .16$^{*}$ | - | | | | | | | | | | |
| 7. Anxiety T1 | .58$^{***}$ | .16$^{*}$ | .10 | .05 | .16$^{*}$ | .52$^{***}$ | - | | | | | | | | | |
| 8. Stress T1 | .65$^{***}$ | .10 | .20$^{**}$ | .17$^{*}$ | .16$^{*}$ | .60$^{***}$ | .63$^{***}$ | - | | | | | | | | |
| 9. Negative affectivity T2 | .81$^{***}$ | .26$^{***}$ | -.10 | .01 | .30$^{***}$ | .53$^{***}$ | .50$^{***}$ | .57$^{***}$ | - | | | | | | | |
| 10. Detachment T2 | .28$^{***}$ | .87$^{***}$ | .02 | -.03 | .39$^{***}$ | .38$^{***}$ | .12 | .14$^{*}$ | .35$^{***}$ | - | | | | | | |
| 11. Dissociality T2 | .03 | .12 | .81$^{***}$ | .42$^{***}$ | -.11 | .07 | .09 | .19$^{***}$ | .04 | .09 | - | | | | | |
| 12. Disinhibition T2 | -.03 | -.01 | .32$^{***}$ | .80$^{***}$ | -.56$^{***}$ | .13 | .02 | .15$^{*}$ | .08 | .04 | .41$^{***}$ | - | | | | |
| 13. Anankastia T2 | .39$^{***}$ | .39$^{***}$ | -.21$^{**}$ | -.52$^{***}$ | .81$^{***}$ | .24$^{***}$ | .22$^{**}$ | .21$^{**}$ | .42$^{***}$ | .40$^{***}$ | -.11 | -.52$^{***}$ | - | | | |
| 14. Depression T2 | .44$^{***}$ | .34$^{***}$ | .00 | .13 | .07 | .64$^{***}$ | .41$^{***}$ | .42$^{***}$ | .54$^{***}$ | .45$^{***}$ | .02 | .16$^{*}$ | .14$^{*}$ | - | | |
| 15. Anxiety T2 | .44$^{***}$ | .15$^{*}$ | .01 | .08 | .09 | .44$^{***}$ | .57$^{***}$ | .38$^{***}$ | .58$^{***}$ | .20$^{**}$ | .06 | .10 | .18$^{**}$ | .57$^{***}$ | - | |
| 16. Stress T2 | .46$^{***}$ | .12 | .13 | .19$^{**}$ | .02 | .46$^{***}$ | .40$^{***}$ | .52$^{***}$ | .60$^{***}$ | .22$^{**}$ | .16$^{*}$ | .20$^{**}$ | .12 | .68$^{***}$ | .65$^{***}$ | - |
| *M* | 35.83 | 27.86 | 23.47 | 24.69 | 40.67 | 6.12 | 3.69 | 6.22 | 35.92 | 27.46 | 23.34 | 24.79 | 40.42 | 5.82 | 3.38 | 6.02 |
| *SD* | 8.21 | 8.33 | 6.13 | 6.38 | 6.67 | 4.68 | 3.74 | 3.98 | 9.04 | 8.55 | 6.56 | 6.38 | 6.71 | 4.87 | 3.71 | 4.02 |
| Cronbach's α | .83 | .84 | .74 | .77 | .78 | .89 | .79 | .80 | .87 | .86 | .78 | .78 | .78 | .91 | .81 | .80 |
| McDonald's ω | .84 | .84 | .74 | .76 | .78 | .89 | .80 | .80 | .88 | .87 | .79 | .78 | .78 | .91 | .82 | .81 |

*Note.* $^{*}p < .05$. $^{**}p < .01$. $^{***}p < .001$.

Table S2

*Results From Three Hierarchical Regressions With T1 Negative Affectivity as Predictor of T2 Psychological Distress Domains Beyond Baseline*

| Variables | Depression | | | Anxiety | | | Stress | | |
|---|---|---|---|---|---|---|---|---|---|
| | β [95% CI] | $R^2$ | $\Delta R^2$ | β [95% CI] | $R^2$ | $\Delta R^2$ | β [95% CI] | $R^2$ | $\Delta R^2$ |
| Step 1 | | $.405^{***}$ | - | | $.323^{***}$ | - | | $.272^{***}$ | - |
| Baseline[a] | $.64^{***}$ | | | $.57^{***}$ | | | $.52^{***}$ | | |
| | [.53, .74] | | | [.46, .68] | | | [.40, .64] | | |
| Step 2 | | $.419^{***}$ | $.014^{*}$ | | $.341^{***}$ | $.018^{*}$ | | $.297^{***}$ | $.025^{**}$ |
| Baseline | $.56^{***}$ | | | $.47^{***}$ | | | $.39^{***}$ | | |
| | [.44, .69] | | | [.34, .61] | | | [.24, .54] | | |
| T1 Negative affectivity | $.14^{*}$ | | | $.16^{*}$ | | | $.21^{**}$ | | |
| | [.01, .27] | | | [.03, .30] | | | [.06, .36] | | |

*Note.* [a] ”Baseline “corresponds to T1 depression for the hierarchical regression predicting T2 depression, T1 anxiety for the hierarchical regression predicting T2 anxiety, and T1 stress for the hierarchical regression predicting T2 stress. β = standardized regression coefficient. 95 % CI = 95% confidence interval. $^{*}p < .05$. $^{**}p < .01$. $^{***}p < .001$.

Table S3

*Results From Three Hierarchical Regressions With T1 Detachment as Predictor of T2 Psychological Distress Domains Beyond Baseline*

| Variables | β [95% CI] | $R^2$ | $\Delta R^2$ | β [95% CI] | $R^2$ | $\Delta R^2$ | β [95% CI] | $R^2$ | $\Delta R^2$ |
|---|---|---|---|---|---|---|---|---|---|
| Step 1 | | .405*** | - | | .323*** | - | | .272*** | - |
| Baseline[a] | .64*** | | | .57*** | | | .52*** | | |
| | [.53, .74] | | | [.46, .68] | | | [.40, .64] | | |
| Step 2 | | .417*** | .012* | | .327*** | .003 | | .276*** | .004 |
| Baseline | .59*** | | | .56*** | | | .52*** | | |
| | [.48, .71] | | | [.44, .67] | | | [.40, .63] | | |
| T1 Detachment | .12* | | | .06 | | | .06 | | |
| | [.003, .23] | | | [-.06, .17] | | | [-.06, .18] | | |

*Note.* [a] "Baseline "corresponds to T1 depression for the hierarchical regression predicting T2 depression, T1 anxiety for the hierarchical regression predicting T2 anxiety, and T1 stress for the hierarchical regression predicting T2 stress. β = standardized regression coefficient. 95 % CI = 95% confidence interval. $^{*}p < .05$. $^{**}p < .01$. $^{***}p < .001$.

Table S4

*Results From Three Hierarchical Regressions With T1 Dissociality as Predictor of T2 Psychological Distress Domains Beyond Baseline*

| Variables | β [95% CI] | $R^2$ | $\Delta R^2$ | β [95% CI] | $R^2$ | $\Delta R^2$ | β [95% CI] | $R^2$ | $\Delta R^2$ |
|---|---|---|---|---|---|---|---|---|---|
| Step 1 | | .405*** | - | | .323*** | - | | .272*** | - |
| Baseline[a] | .64*** | | | .57*** | | | .52*** | | |
| | [.53, .74] | | | [.46, .68] | | | [.40, .64] | | |
| Step 2 | | .406*** | .001 | | .326*** | .003 | | .273*** | .001 |
| Baseline | .64*** | | | .57*** | | | .52*** | | |
| | [.53, .75] | | | [.46, .69] | | | [.40, .64] | | |
| T1 Dissociality | -.03 | | | -.05 | | | .03 | | |
| | [-.14, .08] | | | [-.17, .06] | | | [-.09, .15] | | |

*Note.* [a] "Baseline "corresponds to T1 depression for the hierarchical regression predicting T2 depression, T1 anxiety for the hierarchical regression predicting T2 anxiety, and T1 stress for the hierarchical regression predicting T2 stress. β = standardized regression coefficient. 95 % CI = 95% confidence interval. $^{*}p < .05$. $^{**}p < .01$. $^{***}p < .001$.

Table S5

*Results From Three Hierarchical Regressions With T1 Disinhibition as Predictor of T2 Psychological Distress Domains Beyond Baseline*

| Variables | β [95% CI] | $R^2$ | $\Delta R^2$ | β [95% CI] | $R^2$ | $\Delta R^2$ | β [95% CI] | $R^2$ | $\Delta R^2$ |
|---|---|---|---|---|---|---|---|---|---|
| Step 1 | | .405*** | - | | .323*** | - | | .272*** | - |
| Baseline[a] | .64*** | | | .57*** | | | .52*** | | |
| | [.53, .74] | | | [.46, .68] | | | [.40, .64] | | |
| Step 2 | | .409*** | .004 | | .326*** | .003 | | .283*** | .011 |
| Baseline | .63*** | | | .57*** | | | .50*** | | |
| | [.52, .74] | | | [.45, .68] | | | [.39, .62] | | |
| T1 Disinhibition | .06 | | | .05 | | | .11 | | |
| | [-.05, .17] | | | [-.06, .17] | | | [-.01, .23] | | |

*Note.* [a] ”Baseline “corresponds to T1 depression for the hierarchical regression predicting T2 depression, T1 anxiety for the hierarchical regression predicting T2 anxiety, and T1 stress for the hierarchical regression predicting T2 stress. β = standardized regression coefficient. 95 % CI = 95% confidence interval. $^{*}p < .05$. $^{**}p < .01$. $^{***}p < .001$.

Table S6

*Results From Three Hierarchical Regressions With T1 Anankastia as Predictor of T2 Psychological Distress Domains Beyond Baseline*

| Variables | β [95% CI] | $R^2$ | $\Delta R^2$ | β [95% CI] | $R^2$ | $\Delta R^2$ | β [95% CI] | $R^2$ | $\Delta R^2$ |
|---|---|---|---|---|---|---|---|---|---|
| Step 1 | | .405*** | - | | .323*** | - | | .272*** | - |
| Baseline[a] | .64*** | | | .57*** | | | .52*** | | |
| | [.53, .74] | | | [.46, .68] | | | [.40, .64] | | |
| Step 2 | | .406*** | .001 | | .323*** | .000 | | .275*** | .003 |
| Baseline | .64*** | | | .57*** | | | .53*** | | |
| | [.53, .75] | | | [.45, .68] | | | [.41, .65] | | |
| T1 Anankastia | -.03 | | | .00 | | | -.06 | | |
| | [-.14, .08] | | | [-.11, .12] | | | [-.18, .06] | | |

*Note.* [a] ”Baseline “corresponds to T1 depression for the hierarchical regression predicting T2 depression, T1 anxiety for the hierarchical regression predicting T2 anxiety, and T1 stress for the hierarchical regression predicting T2 stress. β = standardized regression coefficient. 95 % CI = 95% confidence interval. $^{*}p < .05$. $^{**}p < .01$. $^{***}p < .001$.
